# New Approach to Modelling the Impact of Heavy Metals on the European Union’s Water Resources

**DOI:** 10.3390/ijerph20010045

**Published:** 2022-12-21

**Authors:** Monica Laura Zlati, Lucian Puiu Georgescu, Catalina Iticescu, Romeo Victor Ionescu, Valentin Marian Antohi

**Affiliations:** 1 Department of Business Administration, Dunarea de Jos University of Galati, 800001 Galați, Romania; 2 Department of Chemistry, Physics and Environment, REXDAN Research Infrastructure, Dunarea de Jos University of Galati, 800008 Galați, Romania; 3 Department of Administrative Sciences and Regional Studies, Dunarea de Jos University of Galati, 800201 Galați, Romania; 4 Department of Finance, Accounting and Economic Theory, Transylvania University of Brasov, 500036 Brașov, Romania

**Keywords:** water pollution, heavy metals, regional vulnerabilities, public policies, econometric model

## Abstract

The present research aims to address the highly topical issue of heavy metal water pollution from an integrated European perspective, i.e., to quantify through modelling a general model of water pollution reduction in the EU. The objectives of the study are mainly aimed at identifying effective solutions to reduce heavy metal water pollution and providing supranational decision-makers with public policy directions in the field. The research methods consist of the foundation of working hypotheses based on the study of the literature, the consolidation of official statistical databases in the field, econometric modelling and the conceptualisation of a general model and its testing and validation by statistical methods. The results of the analysis consist of the following marginal contributions: the identification of a general model for combating heavy metal pollution; the calculation of the degree of contribution of regional policies to the general model; and the identification of effective solutions to improve the combating of heavy metal water pollution in Europe. The main conclusion of the analysis shows that significant progress has been achieved at the EU level in the field of combating heavy metal water pollution. However, the level of disparity and poor policy coordination are real vulnerabilities for the EU.

## 1. Introduction

### 1.1. General Approach

Water pollution is a global problem as, according to the World Health Organisation (WHO), 2.1 million people do not have access to a safe drinking water source. In the literature, water pollution is contamination with dangerous substances, often chemicals or micro-organisms, that reduce the quality of water so much that it becomes toxic for human consumption or the environment.

Depending on the type of water source, there is the pollution of groundwater, surface water, oceans, agricultural water pollution (through irrigation canals) and wastewater. In terms of pollution sources, groundwater is contaminated with pesticides, fertilisers, septic substances, heavy metals and other sources coming from the interaction of groundwater with various pollution sources (waste–soil interaction, cyanide interaction in the extractive industry soil).

The pollution of surface waters comes from manufacturing industries and productive farms located near waters that cause the aquatic environment to become harmful to aquatic flora and fauna, affecting nutrients in the water through excess nitrates and phosphates or through pollution with fertilisers or waste products from farms.

Another source of surface water contamination is wastewater from human settlements, which increases the toxicity of surface water in the vicinity of these discharges. Waste directly discharged into water is another source of surface water pollution.

Ocean waters are contaminated with chemicals, nutrients and heavy metals, and are secondarily affected by the runoff that flows into them. A major challenge to the ocean and marine aquatic environment is microplastics, which are carried by wind and storms into the world’s oceans and seas and poison the aquatic environment. Oil spills are another source of pollution in ocean waters, along with carbon emission residues from air pollution which reach the oceans via rainfall.

The typology of water pollution aims at assessing toxicity, concentrations, chemical reactions, possibilities of the removal of polluting sources, environmental effects on aquatic flora and fauna, the impairment of biological processes and impact on human health.

According to the OECD [1], the costs of water pollution are significant and are aimed at both treating water for human consumption and reducing contamination with various chemicals in Europe. Belgium has an annual cost of more than $167 million for treating water sources for human consumption. In France, more than $695 million is allocated to limiting nitrate and pesticide emissions from agriculture. In the Netherlands, more than USD 370 million is allocated to stop nitrate and phosphate water pollution, while in Sweden, the costs of eutrophication in coastal waters exceed USD 1257 million, and in the Baltic Sea, USD 719 million. In Europe, on average, the impact on human health and ecosystems due to the nitrogen pollution of rivers and seas is up to USD 164 million, while the health costs of consuming nitrate-infested drinking water due to the costs of treating colon cancer exceed USD 1 billion annually.

### 1.2. Literature Review

In this context, the impact of heavy metals on ecosystem quality and health is significant, having been noted since the early 1990s, when the Air Convention-specific legislation led to reductions in emissions of heavy metals across Europe. As a result of the efforts of the European Commission and the Council, between 2005–2020, lead emissions fell by 49%, mercury emissions by 51% and cadmium emissions by 39% at the EU27 level [2].

The biggest reductions were recorded for lead in Latvia (98%), Malta (95%) and Greece (91%). At the other end of the scale, Poland and Lithuania were the worst performers, with pollution reductions of 9% and 1%, respectively.

As far as mercury is concerned, according to European Environment Agency data [2], there have been increases in pollution in Estonia of 10%; in addition, Lithuania and Latvia have recorded insignificant reductions in mercury pollution of 2% and 6%, respectively. The biggest reductions in pollution were recorded in Bulgaria (−71%), Greece (−70%) and Cyprus (−69%).

Cadmium pollution has increased the most in Malta (15%), Hungary (3%), Lithuania (2%) and Poland (1%) despite sustained efforts to reduce it. The largest reductions were recorded in Greece (−84%), Bulgaria (−75%) and Cyprus (−62%).

**Figure 1:**
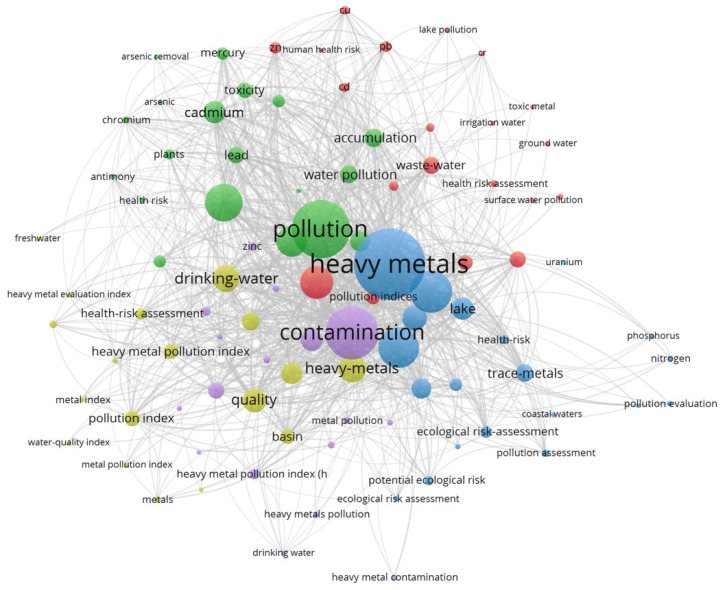
Meta-analysis of areas of interest in the literature on heavy metal water pollution.

The importance of the research topic also results from the frequent approach by specialists, who have investigated both the causes and extent of the heavy metal pollution of water sources and its effects on ecosystems and population health (see Figure 1).

Following a meta-analysis of papers published on the Web of Science platform, according to the criteria of heavy metals pollution in water, a sample of 18,749 articles resulted, of which, 8198 were published in the period 2019–2022, articles that proved to be of real interest in academia, producing a number of 80,000 citations in 47,777 articles, excluding self-citations. The average number of citations per publication is 9.86, and the Hirsch index is 94. The most interesting papers are those in the field of sustainable heavy metal water cleaning technologies, the toxic effects of heavy metal pollution on human health and remediation measures. Other areas of significant interest with over 100 citations per item are waste management in developed cities and conventional and unconventional methods of waste treatment.

After processing the information from the Web of Science platform using the WosViewer program, a map of the co-occurrence of the research items was created, divided into six clusters, the first of which includes the impact of heavy metal pollution (cadmium, chromium, copper, zinc and lead) on groundwater and surface water levels, risk factors on human health and water waste management. Cluster 2 comprises 19 items which address heavy metal accumulations and risks to the health of organisms and plants with the monitoring of sediment and toxicity in soil and water. Cluster 3, which contains 19 items, covers coastal water pollution, environmental risk assessment, nitrogen and phosphorus pollution, the detection of heavy metals in water and water quality. Cluster 4, which contains 18 items, includes research on the degrees of contamination, the quality of drinking water and water for current population consumption, and pollution indices, including a water quality index and a heavy metal pollution index. Cluster 5 (17 items) includes items on groundwater pollution, heavy metal pollution index, pollution sources, zinc pollution and water quality levels. Cluster 6 concerns uranium pollution.

The negative impact of the extractive industry on water resources is analysed by authors Kumar et al. [3] in the context of ensuring ecosystem sustainability. The authors use a multidisciplinary approach to quantify and measure the level of contamination and the degree of ecological and human health risk due to heavy metals in Fiji’s water sources. Analyses of water samples showed serious exceedances of cadmium, lead and mercury concentrations, which are significantly higher than the maximum values laid down in international legislation. The authors use four models for water contamination with cadmium, lead, mercury and nickel. The toxic impact of heavy metals has also been felt in aquatic life. Heavy metal mining is a potential health risk according to Obasi and Akudinobi [4]. As lead, cadmium, arsenic and mercury are highly carcinogenic, assessing the levels of these metals in water resources is becoming an urgent necessity. The authors use tests based on atomic absorption spectrophotometry and ultraviolet/visible spectroscopy. The analysis concludes that heavy metal levels in water sources are high and comply with the relationships: Pb^2+^ > Hg^2+^ > Hg^2+^ > As^2^+ > Cd^2+^ > Mn^2+^ > Ag^2+^ > Se^2+^ > Ni^2+^ > Cr^2+^ > Cu^2+^. All these levels exceed the WHO recommended standard for drinking water. Potential health risks are associated with the accumulation of toxic heavy metals in tissues, including Parkinson’s disease, arsenicosis, acrodynia, selenosis, Alzheimer’s disease, hair loss, mental imbalance and miscarriage in women. The same topic is addressed by authors Santana et al. [5], who consider water resources to be the most vulnerable environmental element to the direct impact of mining. Analyses of water sources revealed that there is significant contamination with Cd, Pb and U, above the limits set by international regulatory legislation. The authors believe that high concentrations of heavy metals in water sources are mainly related to mining activities and, to a lesser extent, to natural sources. Furthermore, based on the analysis of sediment quality indices (TEL, PEL, ΣTU and PEL-Q), it was shown that the probability of heavy metals inducing adverse toxic effects on aquatic organisms is 25%. An investigation of the effects of mining on water resources in adjacent areas was carried out by Igwe et al. [6] using atomic absorption spectrophotometry and covering a mining region in Nigeria. The results of the analyses confirm different values of heavy metal water pollution, as follows: Cd (0.18–4.37), Pb (0.06–10.11), Zn (0.13–7.11), Ni (0.02–1.21), Mn (0.04–1.16), Fe (0.03–2.04) and Cr (0.02–0.48) in the case of surface waters, and Cd (0.02–2.00), Pb (0.16–3.18), Zn (0.13–5.16), Ni (0.01–1.54), Mn (0.01–2.17), Fe (0.01–2.50) and Cr (0.01–0.28) for groundwater. The authors conclude that the deterioration of water resources through mine drainage prevents drinking water supply in the studied areas and is likely to have also affected adjacent regions.

Other authors such as Jafarzadeh et al. [7] point out that groundwater aquifers are the second most abundant source of drinking water worldwide and that their pollution with heavy metals causes toxic effects that are difficult to quantify. The study under review focuses on underground water sources in Iran and looks at three heavy metals (chromium, cadmium and lead). In order to calculate human non-cancer values, the authors use the Hazard Quotient (HQ) calculation and the Monte Carlo simulation method with 10,000 repetitions. The studies are conducted on a sample comprising four age groups (adults, adolescents, children and infants). Sensitivity analysis showed that the concentration of heavy metals in drinking water presents the highest carcinogenic risk, and the authors propose that decision-makers in public administration should be more widely involved in making adequate measurements of heavy metal concentrations in groundwater.

A topical challenge is the treatment of heavy metals. One method used for this purpose is the graphene oxide nanocomposite coated with folic acid (FA-GO), as stated by Eftekhari et al. [8]. According to the authors, graphene oxide (GO) is modified with folic acid (FA) to synthesise the FA-GO nanocomposite, which virtually absorbs heavy metals from water.

Sources of heavy metal drinking water pollution and human health risks associated with drinking water resources were assessed by authors Egbueri and Mgbenu [9], with reference to a region in Nigeria. The health hazard index calculated by the authors indicates a 25% chance of adults and children contracting serious diseases or developing various forms of cancer. It has thus been shown that there is a strong correlation between heavy metals in water and natural and anthropogenic processes, lead being considered the main pollutant. Studies also confirm that water from hand-dug wells and deeper boreholes is less contaminated and therefore more suitable for drinking than water from springs, streams and shallow wells.

The presence of heavy metals in Iranian drinking water resources was quantified by authors Ravanipour et al. [10] based on a complex meta-analysis covering PubMed, Web of Science, EMBASE, Scopus, Google Scholar databases and over 1100 articles and studies in the field. The results of the analysis reveal that the group average concentration level is Pb (37.22) > Hg (4.49) > Cd (4.19), above WHO standards.

With the implementation of sustainable economic development, concerns about the quality of the environment, including water resources, have increased. In this context, the authors Priya et al. [11] use biosorption as a method to remove pollutants from water resources. This study investigates the occurrence of heavy metals and their removal by biosorption techniques at temperatures between 20 and 35 °C.

In the context of implementing the circular economy in wastewater treatment, authors Rey-Martínez et al. [12] compare the results obtained for heavy metals with the closest possible regulatory framework. These results support the idea of the market penetration of recovered products and the need for a new regulatory framework for these products that is appropriate for their current uses. The analysis of heavy metals in 15 recovered products is carried out by the authors Rey-Martínez et al. [12]. This analysis concludes that in the case of the food industry, heavy metals slightly exceeded the limits for very specific pollutants and only for a specific use. As a result, the authors argue the need for a new regulatory framework for these recovered products to match their current uses. Other authors, such as Camilleri [13], review the latest European environmental policies, including “new circular economy plans for a cleaner and more competitive Europe”. The author conducts a meta-analysis focusing on the circular economy in the EU context. The same author offers solutions to national and supranational decision-makers on “planning, organising, successfully implementing and measuring circular economy practices for sustainable supply chains in Europe”.

Heavy metal water contamination was quantified by authors Gayathri et al. [14] using multivariate techniques and environmental indices. The tests carried out were compared with the maximum permissible limit values recommended by the World Health Organization. The order of heavy metals found in the sediment was: Pb > Mn > Ni > Zn > Cr > Cu > Co and was the basis for the production of maps based on the Geographic Information System.

Activities such as energy production, metal and chemical manufacturing and waste and wastewater management cause chromium contamination of water sources. As authors Tumolo et al. [15], point out, at the EU level, the discharge of chromium into waters is subject to national guidelines, which vary depending on the type of industry and receiving water body. Attention is drawn to hexavalent chromium compounds because of their toxic effects on humans, animals, plants and micro-organisms. The risks of chromium pollution of water resources range from skin irritation to AND and cancer development.

The issue of sustainable water resources management in the EU is addressed by Farmaki et al. [16] in the context of the “Fair Transition Plans” in the European Union. The analysis focuses on the region of Macedonia in Greece and aims on the one hand at analysing the literature, sources from the government’s core strategies, as well as policy and decision documents, and on the other hand, at formulating research questions by synthesising relevant data. The authors offer solutions for improving the management of sustainable water resources management in line with EU water policy. Greece’s water resources are the subject of another approach by Kourgialas [17], who starts in his analysis from the reality that Greece’s scarce water resources show great regional disparity and are under the impact of climate change, pollution and high agricultural use.

The digitisation environment nexus provides empirical evidence on the influence of digital transformation on environment and sustainability in the European Union according to the authors Ha et al. [18]. The authors use a statistical database covering 25 European countries over the period 2015–2020 and quantify the impact of digitisation on human health and ecosystem protection. The first conclusion of the analysis is that the digital transformation process improves environmental performance, including water resources. Moreover, the digital transformation process is found to have negative effects in the short term but positive effects in the long term. The analysis carried out targets the quantitative and qualitative aspects of water resources in Greece for each river basin and follows the efficiency of water use, ensuring the sustainability of water resources in Greece. Insular Greece (island of Crete) is under consideration in the context of developing appropriate water governance regulations that promote the development of integrated water management plans while allowing flexibility in water use. The authors Tzanakakis et al. [19] consider that solutions for water resources management should cover: “the use of alternative water sources (treated water and brackish water), efficient water use, pricing policy reform, effective water control and management, and investment in research and innovation to support the above actions”.

Under the European Drinking Water Directive, reduced thresholds for heavy metals in domestic drinking water have been introduced. The authors García-Miranda Ferrari et al. [20] give as an example the threshold for the concentration of lead ions (Pb^2+^) in drinking water, which has been reduced from 10 to 5 parts per billion (ppb). The authors review electrochemical methods, materials and electrode modifications that have the potential to underpin a new generation of portable electrochemical sensors capable of quantifying heavy metal concentrations in water at the trace ion level.

An interesting approach to water resources is the one carried out by the authors Berto et al. [21] which focuses on the Adriatic–Ionian region (ADRION region). The study is carried out in accordance with EU Directives (WFD—Water Framework Directive, MSFD—Marine Strategy, Marine Strategy Framework Directive) and the Barcelona Convention Protocols which aim to assess pollution levels and prevent and/or mitigate the impact of water pollution on the marine environment. The review covers six EU and non-EU countries along the Adriatic and Ionian Seas and concludes with a methodological proposal to define a common protocol for assessing the metal contamination of seawater, sediments and biota. The authors consider that the harmonised assessment of heavy metals at the national, regional and sub-regional levels is a challenge to ensure the best level of protection of the coastal and marine environment in the region under consideration. A similar approach, targeting the Mediterranean and Adriatic seas, is taken by authors Molina Jack et al. [22], who focus on monitoring heavy metals in water sources. The authors support international cooperation in the field, the creation of common metadata and data formats, and standard vocabularies to ensure homogeneous syntax and semantics.

The transfer of urban pollutants such as heavy metals into the consumer food chain from the perspective of urban green oases is analysed by authors Ziss et al. [23]. The authors focus on the potential risk of heavy metal contamination in the context of urban gardening. The research covers urban areas in Austria and concludes that traces of soil metals (Pb, Cd, Zn) in urban gardens exceeded the limits recommended by this country.

Authors such as Kanstrup and Thomas [24] analyse the consequences of externalising the effects of spent lead ammunition on society. The analysis is presented in the context of the European Commission’s move to introduce fundamental restrictions on the use, marketing and possession of lead ammunition for all types of hunting and target shooting. This would reduce the lead infestation of game, including aquatic wildlife, and provide additional health protection and a safe source of venison products for consumers.

The state of soil biodiversity in the context of the new European Soil Programme is analysed by the authors Köninger et al. [25]. The authors conduct a meta-analysis of 507 literature references related to regulation, incentives and knowledge and strategic policy documents at the EU and national levels on soil pollution. The authors point out that at the EU level, only eight member states explicitly address threats to soil biodiversity in regulatory instruments. Another 13 member states focus mainly on implicit threats to soil biodiversity, whereas six countries do not consider soil biodiversity.

One source of heavy metal pollution is the poor management of municipal solid waste. According to the authors Mazzucco et al. [26], critical situations such as the one triggered in western Sicily by fires in landfills may be sources of heavy metal pollution. Heavy metal levels above legal limits were detected in upper and lower soil samples, posing contamination risks to water sources in the region. The theme of sustainability of the waste management system in Italy is taken up by the authors Di Maria et al. [27] based on an analysis covering the period 2007–2016 and focusing on the Integrated Sustainability Indicator (ISI).

The impact of heavy metals on human health is addressed by the authors Elonheimo et al. [28] based on the connection between osteoporosis and the body burden of heavy metals such as cadmium (Cd) and lead (Pb). The impact of heavy metals on human health is also analysed from the perspective of seafood consumption by authors Ramon et al. [29]. The authors aim to measure the levels of arsenic, mercury, cadmium and lead in the raw tissues of seafood from the eastern Mediterranean Sea. The analysis is based on a questionnaire of 296 samples from 11 different seafood species. The results of the analysis revealed that total arsenic concentrations were significantly higher in crustaceans and cephalopods than in fish. Cadmium was detected in a third of the samples, and lead was detected in eight samples.

Water resources must meet safety and security criteria. Starting from the new European Drinking Water Directive issued on 12 January 2021, the authors Dettori et al. [30] mention the novel elements that have emerged in relation to the risk-based approach, the updating of some quality standards, the identification of possible emerging pollutants in water supplies, etc. The authors mention that this directive is the first European legislation adopted following a European Citizens’ Initiative.

From the new technologies’ (nano-technologies) point of view, there have been significant improvements in the analysis and removal of heavy metals from water resources. These techniques refer to electrochemical, colourimetric, fluorescent and biosensor technology. According to the authors Gong et al. [31], new nano-technologies have efficiency advantages in treating water resources and detecting and removing heavy metals from these resources. Another technology aimed at removing heavy metals from water sources is based on magnetic materials. According to the authors Hojjati-Najafabadi et al. [32], this technology is effective in controlling and monitoring heavy metals in water. The authors present observations and future predictions on magnetic nanosensors for the control of hazardous pollutants in water resources and environmental applications.

From an economic point of view, the authors Abu Hasan et al. [33] consider that the cheapest procedure to treat water sources is the biological one. These treatment technologies have the ability to treat contaminants in polluted drinking water supplies, such as endocrine-disrupting heavy metals and microbial contaminants.

Other authors, such as Saleh et al. [34], question the recalcitrance of heavy metals in wastewater. The authors conduct a review of heavy metal removal technologies from water sources, including chemical precipitation, photocatalysis, flotation, ion exchange, remediation, electrochemistry, membrane technologies and coagulation/flocculation.

The negative impact of heavy metals on water resources is quantified based on a meta-analysis by the authors Nazaripour et al. [35]. The authors note that the number of articles on this topic increased between 2000 and 2019 by 1700%. The studies focused primarily on technical processes such as adsorption, membrane filtration and ion exchange. Efficiency, environmental impacts and cost-effectiveness were chosen as criteria to compare these methods, with adsorption coming first.

Heavy metal water pollution detection and remediation technologies are the subjects of a study by authors Jain et al. [36], from the perspective of using imaging solutions. The authors consider several technologies such as transduction techniques (colourimetric and photoluminescence), sensor materials, readout instruments and sampling methods.

Another method of measuring the degree of heavy metal contamination of water is Fourier Transform Infrared Spectroscopy (FT-IR). The authors Mamera et al. [37] use FT-IR calibration for the metals Ag, Cd, Cu, Pb and Zn. The results were compared with atomic absorption spectrometer (AAS) measurements and targeted surface and groundwater sources in South Africa.

There is a clear involvement of industry in increasing the concentration of heavy metals in water resources according to Dhiman and Kondal [38]. The authors propose adsorption as a widely used method to remove these non-biodegradable heavy metals. This method is capable of absorbing various heavy metal ions such as Cd^2+^, Hg^2+^, As^3+^, Pb^2+^, Cr^6+^, Ni^2+^, Co^2+^ and Cu^2+^. In the same context, authors R. Kumar et al. [39] investigate the efficiency of the adsorption method of heavy metals arsenic, lead, chromium and selenium on graphene-based magnetic plates. The authors also refer to the need to ensure sustainability based on new functionalised magnetic compounds based on magnetised graphene.

Other authors Xiang et al. [40] propose another technical solution for the treatment of heavy metal-polluted water, namely, the membrane separation method, which is characterised by high efficiency, easy operation and low space requirements.

There are concerns about heavy metal water pollution from mining in non-EU European countries. One such study by the authors Sadiku et al. [41] aims to quantify the impact of the Artana mine on the heavy metal pollution of the Marec River. The authors use the analysis standards ISO 5667-6 for surface water and ISO 5667-11 for groundwater. The determination of heavy metal concentration in water was carried out with the SAA method (Atomic Absorption Spectrophotometry). The results of the analyses showed significant exceedances of heavy metal concentrations in the studied industrial area. Heavy metal water pollution due to mining is approached from a different perspective by the authors Kadriu et al. [42], who focus on the pollution of the urban environment. The use of standard analytical methods was accompanied by the ICP–OES (Inductively Coupled Plasma–Optical Emission Spectrometry) measurement technique to quantify the concentration of heavy metals. An interesting conclusion of the study links the concentration of heavy metals in water to climate change.

A requirement of water source quality maintenance is that the water manager is required to detect most polluting discharges to the sewer. This approach is in line with Directive 2000/60/EC and is also supported by relevant expert studies according to the authors Sambito et al. [43]. The authors perform a meta-analysis in the sense of inserting new information beyond the network topology that they apply in the case of Palermo’s (Italy) sewerage under-capacity.

In relation to water distribution, authors such as Oliker and Ostfeld [44] propose a deterministic optimisation model for maximizing the monitored volume within network clusters, based on fictitious and mobile sensors. The analysis concludes that the simultaneous use of fixed and mobile sensors allows the much more accurate monitoring of water quality.

Accidental contamination and the deliberate injection of toxic agents into water sources are the subjects of research by Piazza et al. [45], who support the need for the widespread use of monitoring sensors. The analysis is supported by the NSGA-II genetic algorithm, which has been coupled with a new diffusive–dispersive hydraulic simulator. An interesting conclusion of the analysis is that the incorrect positioning of water quality sensors leads to inefficient monitoring networks.

### 1.3. Aim and Tusks of the Research

Based on the literature review, we define the following research objectives:

O1. Identifying the dynamics of the heavy metal pollution of European waters by analysing the reports of the European Environment Agency for the period 2000–2021.

O2. The definition of a general spatial and temporal model of combating the pollution of inland, coastal and marine waters with heavy metals.

O3. Analysis of the impact of unsound policies to reduce heavy metal water pollution on the implementation of sustainable development at the European level.

O4. Creating a general framework of vulnerabilities in combating heavy metal pollution in Europe.

O5. Policy proposals to improve water pollution control measures by strengthening them at the European level.

Compared with the existing literature, this article may have these marginal contributions:

(1) To identify the general pattern of combating heavy metal pollution in the EU in a scientific manner based on official statistical reports;
(2) Calculating the degree of contribution of regional policies to the overall model and mapping the levels of public policy disparities in the field at the European level;
(3) Identifying effective solutions to strengthen the fight against heavy metal water pollution in Europe.

It is important to conduct this study because there is a pressing need for new approaches to heavy metal water pollution as its impact on the environment and the health of aquatic systems and the population is significant, with effects that are difficult to reverse in the long term.

## 2. Materials and Methods

In our research, we aimed to analyse the policy to reduce heavy metal pollution of water resources at the EU level through a critical analysis of the dynamics of heavy metal pollution indices of EU waters over the period 2000–2021 using data on water quality in inland, coastal and marine waters reported by countries within WISE SoE reporting [46].

The hypotheses of the present study are defined as follows:

**H1.**  *Combating heavy metal water pollution has a permanent character, but a disproportionate intensity at the EU level depending on the environmental and economic specificity of the member state implementing the policy. This hypothesis is also supported by the research undertaken by the authors Eftekhari et al., Egbueri and Mgbenu, Jafarzadeh et al. and Ravanipour et al. [7,8,9,10].*

**H2.**  *The level of heavy metal water pollution depends on the economic orientation and level of urban development of each member state. The hypothesis is based on research conducted by the authors Camilleri, Gayathri et al., Igwe et al., S. Kumar et al., Obasi and Akudinobi, Priya et al., Rey-Martínez et al., Santana et al. and Tumolo *et al.* [3,4,5,6,11,12,13,14,15].*

**H3.**  *Under conditions of the uniform implementation of environmental policies in the field, the level of pollution in each member state tends to be reduced by at least the difference in disparity compared to the initial moment of non-uniformity. This hypothesis is based on studies carried out by the authors Berto et al., Farmaki et al., García-Miranda Ferrari et al., Ha et al., Kourgialas, Molina Jack et al., Tzanakakis et al. and Ziss *et al.* [16,17,18,19,20,21,22,23].*

From the database containing reports for the last 22 (2000–2021) years of pollution levels with different substances, we extracted information on lead, cadmium and mercury pollution, which have a major impact on the health of the population and on the health of aquatic ecosystems. The indicators used in the analysis are presented in Table 1.

The data were consolidated through the XL program using the panel consolidation technique by the collection period and reporting member state, resulting in a homogeneous database containing a total of 3749 records of the year and country averages of the heavy metal pollution of inland, coastal and marine waters.

The method used is based on the technique of preferential similarity ordering, which involves identifying a general pattern based on particular patterns by correlating the pollution reduction results of one element (cadmium) in relation to the other two elements (mercury and lead). The method is based on the process of classifying and standardizing the matrix observations and calculating the proportions of the contribution of the particular model to the overall pollution output. In mathematical terms, the methodology for calculating system entropy is:

Either W matrix of the spatial and temporal pollution of inland, coastal and marine waters with heavy metals:

(1) $$W=\left(w_{ij}\right)$$

**W**—spatio-temporal pollution indicator, with *i* ϵ [1,27] and is the spatial marker of pollution; *j* ϵ [1,21] is the temporal marker of heavy metal pollution.

We say that $\left(∋\right)\;w_{ij}\neq 0$ that satisfies the criteria of contributing to the general optimal model if and only if:

(2) $$\underset{j\to \infty }{\lim}\frac{w_{ij}-min\left(w_{ij}\right)}{max\left(w_{ij}\right)-min\left(w_{ij}\right)\;}=\frac{\sum_{i=1}^{27}\left(\frac{\sum_{j}\left(w_{ij}\right)}{j}\right)}{i}$$

To determine the coefficients of the pollution matrix, we applied multiple linear regression, establishing the dependent variable cadmium pollution and its components, the regression coefficients being lead and mercury pollution. We obtained the regression equations below:

(3) $$\left\{ \begin{array}{cc} & \text{ ATMeanCADMIUM }=0.049*\text{ ATMeanLEAD }+0.432*\text{ ATMeanMERCURY }\\ & \text{ BAMeanCADMIUM }=0.051*\text{ BAMeanLEAD }+0.679*\text{ BAMeanMERCURY }\\ & \text{ BEMeanCADMIUM }=0.189*\text{ BEMeanLEAD }+1.557*\text{ BEMeanMERCURY }\\ & \text{ BGMeanCADMIUM }=-0.619*\text{ BGMeanLEAD }+4.946*\text{ BGMeanMERCURY }\\ & \text{ CYMeanCADMIUM }=-0.003*\text{ CYMeanLEAD }+0.483*\text{ CYMeanMERCURY }\\ & \text{ CZMeanCADMIUM }=0.433*\text{ CZMeanLEAD }+0.190*\text{ CZMeanMERCURY }\\ & \text{ DEMeanCADMIUM }=0.763*\text{ DEMeanLEAD }+1.343*\text{ DEMeanMERCURY }\\ & \text{ EEMeanCADMIUM }=-0.072*\text{ EEMeanLEAD }+2.453*\text{ EEMeanMERCURY }\\ & \text{ ELMeanCADMIUM }=0.077*\text{ ELMeanLEAD }+0.334*\text{ ELMeanMERCURY }\\ & \text{ ESMeanCADMIUM }=1.690*\text{ ESMeanLEAD }-0.553*\text{ ESMeanMERCURY }\\ & \text{ FIMeanCADMIUM }=0.072*\text{ FIMeanLEAD }+0.196*\text{ FIMeanMERCURY }\\ & \text{ FRMeanCADMIUM }=0.080*\text{ FRMeanLEAD }+2.201*\text{ FRMeanMERCURY }\\ & \text{ HRMeanCADMIUM }=0.052*\text{ HRMeanLEAD }-0.264*\text{ HRMeanMERCURY }\\ & \text{ HUMeanCADMIUM }=0.149*\text{ HUMeanLEAD }-0.057*\text{ HUMeanMERCURY }\\ & \text{ IEMeanCADMIUM }=0.331*\text{ IEMeanLEAD }+1.014*\text{ IEMeanMERCURY }\\ & \text{ ITMeanCADMIUM }=0.126*\text{ ITMeanLEAD }+0.193*\text{ ITMeanMERCURY }\\ & \text{ LTMeanCADMIUM }=0.009*\text{ LTMeanLEAD }+0.321*\text{ LTMeanMERCURY }\\ & \text{ LUMeanCADMIUM }=-0.034*\text{ LUMeanLEAD }+0.413*\text{ LUMeanMERCURY }\\ & \text{ LVMeanCADMIUM }=0.059*\text{ LVMeanLEAD }-0.023*\text{ LVMeanMERCURY }\\ & \text{ MTMeanCADMIUM }=-0.017*\text{ MTMeanLEAD }+12.623*\text{ MTMeanMERCURY }\\ & \text{ NLMeanCADMIUM }=-0.032*\text{ NLMeanLEAD }+6.577*\text{ NLMeanMERCURY }\\ & \text{ PLMeanCADMIUM }=0.148*\text{ PLMeanLEAD }-4.645*\text{ PLMeanMERCURY }\\ & \text{ PTMeanCADMIUM }=0.600*\text{ PTMeanLEAD }-1.461*\text{ PTMeanMERCURY }\\ & \text{ ROMeanCADMIUM }=0.105*\text{ ROMeanLEAD }+0.289*\text{ ROMeanMERCURY }\\ & \text{ SEMeanCADMIUM }=0.030*\text{ SEMeanLEAD }-2.813*\text{ SEMeanMERCURY }\\ & \text{ SKMeanCADMIUM }=0.067*\text{ SKMeanLEAD }-0.564*\text{ SKMeanMERCURY }\\ & \text{ XKMeanCADMIUM }=0.007*\text{ XKMeanLEAD }+0.271*\text{ XKMeanMERCURY } \end{array}\right.$$

From Equation (3), it results that there is a great disparity in the correlation between cadmium and lead pollution reduction policies at the member state level; in some countries, these two elements are treated inversely proportionally (Bulgaria, the Netherlands, Malta and Luxembourg), whereas in countries such as Estonia or Portugal, the strategies are unified, i.e., there is coherence in environmental policies regarding the elimination of both metals through the same actions. At the sample level, the average level of correlation of policies to reduce heavy metal pollution in water is 16%, with most member states recording directly proportional results in terms of combined pollution efforts for both elements.

If for the correlation studied for cadmium and lead, the effects of the combined policies fall within a range of variation of maximum amplitude of 2 [−0.6, 1.69], in the case of the correlation of cadmium and mercury pollution reduction policies, the level of disparity is much wider, the range of variation being 17 [−4.6, 12.6]. There are strong inverse proportional correlations of cadmium and mercury pollution policy implementation in countries such as Poland, Portugal and Sweden, whereas in Malta, the Netherlands, Bulgaria and France, the direct proportional correlation is strong and homogeneously represented for the studied period. This information is presented in Table 2.

The application of econometric modelling allowed the determination of correlation indicators and standard errors associated with the regressions applied to each member state on the data set reported for the period 2000–2021 on inland, coastal and marine waters heavy metal pollution. The study of one-tailed critical probability tests of the correlation values of the three elements showed that the most effective combined pollution control measures for all three elements were applied during the period under review in Austria, Bulgaria, Estonia, Spain, Finland, Hungary, Luxembourg, Portugal and Denmark. The average level of statistical representativeness of the projected modes for these countries was 92.4%. At the other end of the scale, Slovenia, the Czech Republic, Croatia, Italy, Romania and Latvia performed the worst. The average level of statistical representativeness for these countries of the combined pollution control models was 56.9%.

## 3. Results

**Figure 2:**
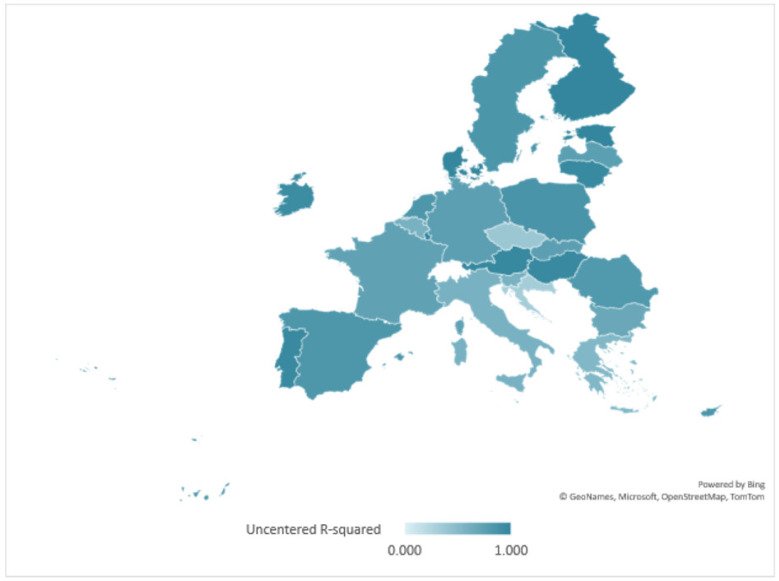
Geospatial distribution of the statistical significance of combined policies to reduce heavy metal pollution in water.

In Figure 2, the map of the geospatial distributions of the statistical significance level of the national econometric models for the combined control of heavy metal pollution in water is shown.

**Figure 3:**
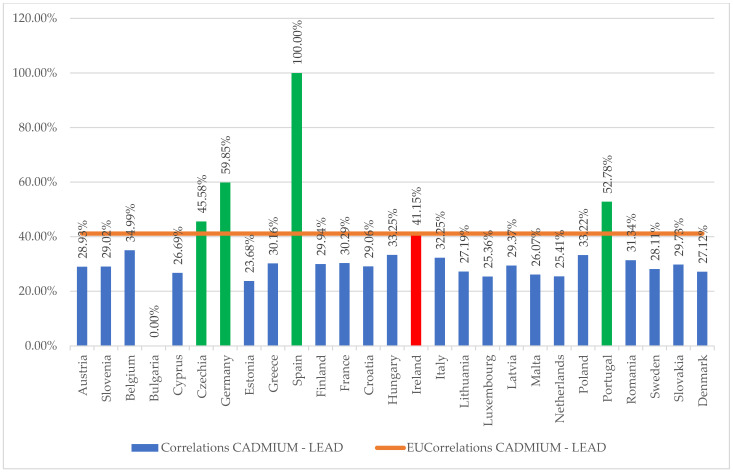
Evaluation of the degree of contribution of the particular model to the overall output of cadmium and lead water pollution control.

The analysis of the spatio-temporal pollution matrix (Equations (1) and (3)) was carried out from the perspective of determining the proportions of the contribution to the general model (Equation (2)), resulting in a dispersion of the cadmium-lead correlation results of 70%, the average degree of contribution being 33.72%, and the homogeneity point of the model for drawing the general model being selected at 40% impact intensity of the correlation level compared to the general dispersion of the sample (see Figure 3).

From Figure 3, it appears that the German, Czech, Spanish, Portuguese and Irish models fit as a contributing principle to the analysed direction (reduction in cadmium and lead pollution) for general standardisation at the EU level.

**Figure 4:**
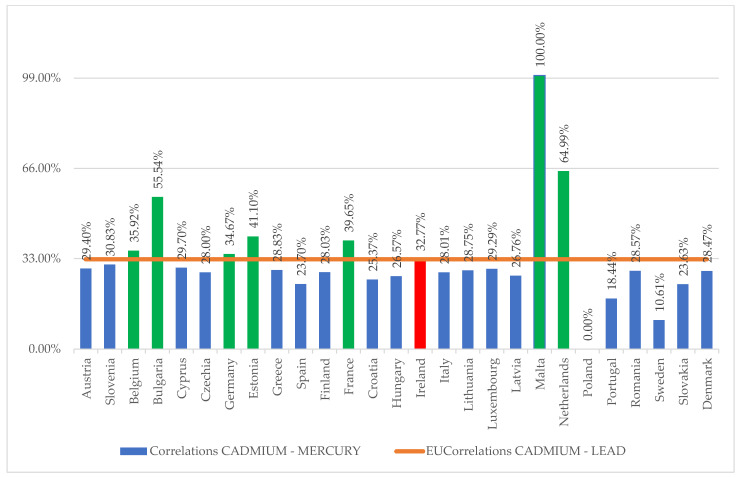
Evaluation of the degree of contribution of the particular model to the overall output of cadmium and mercury water pollution control.

The analysis of the spatio-temporal pollution matrix (Equations (1) and (3)) was carried out from the perspective of determining the proportions of the contribution to the general model (Equation (2)), resulting in a dispersion of the cadmium–mercury correlation results of 68.5%, the average degree of contribution being 32.5%, and the homogeneity point of the model for drawing the general model being selected at 33% impact intensity of the correlation level compared to the general dispersion of the sample (see Figure 4).

From Figure 4, it appears that the Bulgarian, Belgian, German, Estonian, French, Maltese and Dutch models fit as a contributing principle on the analysed direction (reduction in cadmium and mercury pollution) for general standardisation at the EU level.

The comparative analysis of the two figures ranks the German and Irish models for standardisation, with the caveat that the Irish model comes closest to the standardisation parameters selected for the general model. This results in the following general model for reducing heavy metal pollution in water at the EU level:

(4) EUMeanCADMIUM=0.331∗·EUMeanLEAD+1.01·EUMeanMERCURY

The model has an average statistical representativeness of 93.4% applicable to the 22 years analysed, and the values of the statistical tests are centralised in Table 3.

From Table 3, the results show that the mean value of the correlation of pollution control policies is 34%, with a standard error of the regression of 11.3% and a *p*-value of the P-test of model significance (1.67 × 10^−12^) lower than the chosen significance level (0.05), which classifies the general model as representative of the studied phenomenon. Residue normality distribution and heteroscedasticity tests validate the absence of heteroscedasticity and the normal distribution of errors, which leads to the conclusion that in the EU, policies to combat heavy metal water pollution are predominantly oriented towards the isolation of elements with carcinogenic potential.

**Figure 5:**
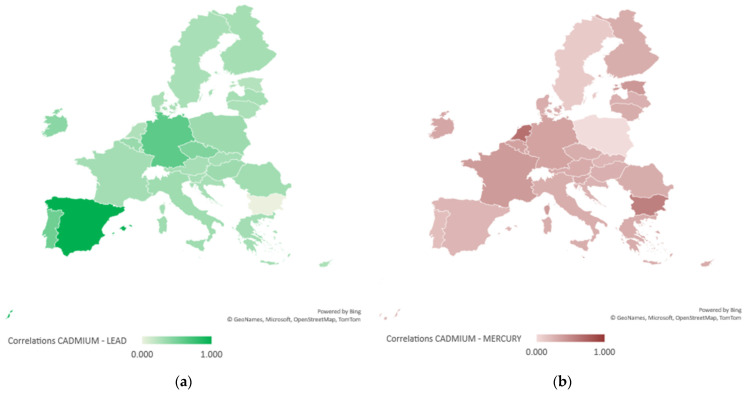
Dispersion of general model correlations by evaluating national models: (**a**) Dispersion for cadmium-lead pollution reduction policies; (**b**) Dispersion for cadmium–mercury pollution reduction policies.

The dispersion of the correlations of the general model by evaluating the national models is shown in Figure 5.

Through modelling, the following working hypotheses were demonstrated:

In the EU, at the member state level, as a result of the efforts of the European Commission and the Council, between 2005–2020, lead emissions decreased by 49%, mercury emissions by 51% and cadmium emissions by 39% at the EU27 level. [2]. There are significant disparities identified during the research and mapped in Figure 5, which supports hypothesis H1. Combating heavy metal water pollution has a permanent character, but a disproportionate intensity at the EU level depending on the environmental and economic specificity of the member state implementing the policy.

According to official reports of the European Environment Agency [47], the level of heavy metal water pollution during the period under review decreased in Belgium, Bulgaria, France, Luxembourg, Portugal and Romania (by more than 50%). These countries benefit from advanced industrial technologies (Belgium, France and Luxembourg) and/or reduced industrial activities (Bulgaria, Portugal and Romania). At the opposite end of the spectrum are countries such as Croatia, Cyprus, Greece, Hungary and Slovenia, where heavy metal water pollution increased by more than 20% during the period under review, due to the use of less developed industrial technologies and seasonal urban agglomerations due to international tourism. These developments demonstrate the H2 hypothesis. The level of heavy metal water pollution depends on the economic orientation and the level of urban development of each member state.

**Figure 6:**
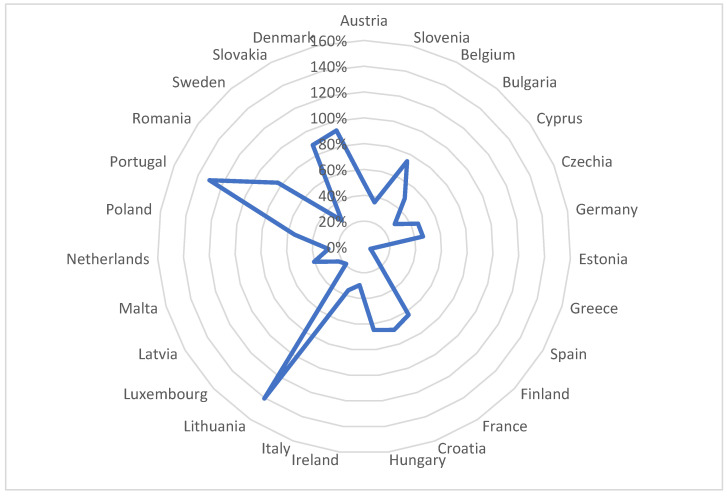
Disparities in policies to combat heavy metal water pollution in the EU.

From the modelling carried out, it emerged that at the EU level, there are large disparities in the strengthening of policies to combat heavy metal water pollution, the level of disparity varying from the calculation of the standard deviation of the average pollution indices in the geospatial dust matrix W between 5% and 140% (see Figure 6).

This information correlated with the results of the national regression models from the pollution bases (Equation (3)) allowed the consolidation of a core of the most uniform policies applied at the EU level, for which the level of disparity varies from the calculation of the standard deviation of the average pollution indices in the geospatial dust matrix W between 13% and 50% (Austria, Czechia, Finland, Italy, Ireland, Luxembourg and Latvia). This demonstrates that by applying strengthened policies to reduce heavy metal water pollution, efficiency can be improved by at least 20%, i.e., from an overall dispersion of 50% to a dispersion of 30%. This aspect demonstrates hypothesis H3. Under conditions of the uniform implementation of environmental policies in the field, the pollution level in each member state tends to be reduced at least by the difference in disparity compared to the initial moment of non-uniformity.

## 4. Discussion

The following aspects on the issue of heavy metal water pollution emerged from the research. Pollution-related risks are a key global environmental issue. Elements such as Cd, Cr, Pb, Ni, Mn and Zn are considered potential heavy metals (HM) in aquatic and terrestrial environments due to their non-degradable and toxic existence. The deterioration of water resources has raised the interest of researchers from the perspective of risks to human health and the environment. The results of a meta-analysis-based study by authors Ravanipour et al. [10] highlight the wide range of variation in concentrations for different pollutants (between 0 and 100) across the three types of water resources (drinking, groundwater and surface). In the case of the heavy metals analysed by us, the authors found concentrations such as: Pb (37.22) > Hg (4.49) > Cd (4.19). The average concentration levels for Pb and Cd were higher than WHO standards.

Heavy metals have specific characteristics (bioaccumulation, long persistence in the natural environment and toxic effects) and are real threats in groundwater contamination. In this way, the very sustainable development of contemporary society is affected. As a result, biosorption is considered to be an effective method of reducing heavy water pollution. This method is recommended by the research of the authors Priya et al. [11].

Water pollution with heavy metals can damage the brain, kidneys and stomach and can lead to death. There is a strong correlation between heavy metal pollution and various forms of cancer. However, the analyses carried out led to some contradictory results. If, according to USEPA—the United States Environmental Protection Agency—Cd is carcinogenic only by inhalation and Pb has no carcinogenic effects, according to Mazzucco et al. [26], lead, cadmium and mercury are highly carcinogenic, and they are toxic according to the authors Elonheimo et al. [28]. The health hazard index afferent to heavy metal water pollution is calculated by the authors Dobrowolski et al. [48], who show that Pb is the priority pollutant affecting water quality.

The pollution of water reservoirs with Cd and Pb may be due to the use of fertilisers in agriculture according to Di Maria et al. [27]. Another source of heavy metal pollution is mining, a fact highlighted by the authors Rodríguez-Espinosa et al. [49].

From the circular economy point of view, wastewater treatment plants should be transformed into water recovery plants by applying new recovery technologies. Akyol et al. [50] state that such recovery technologies have already been tested and implemented, but point out that inadequate heavy metal concentrations can affect health and the environment. The maximum permissible concentration of heavy metals in the soil must comply with the official provisions in this field, such as: the directive issued by the Food and Agriculture Organization (FAO) [51] and the European Directive 86/278/CEE [52]. The same issue of the presence of heavy metals in soil is also treated by other authors [53,54]. In the case of wastewater, the analysis was carried out by [55]. The risks associated with soil and water contamination with heavy metals have been analysed by [54,56].

**Figure 7:**
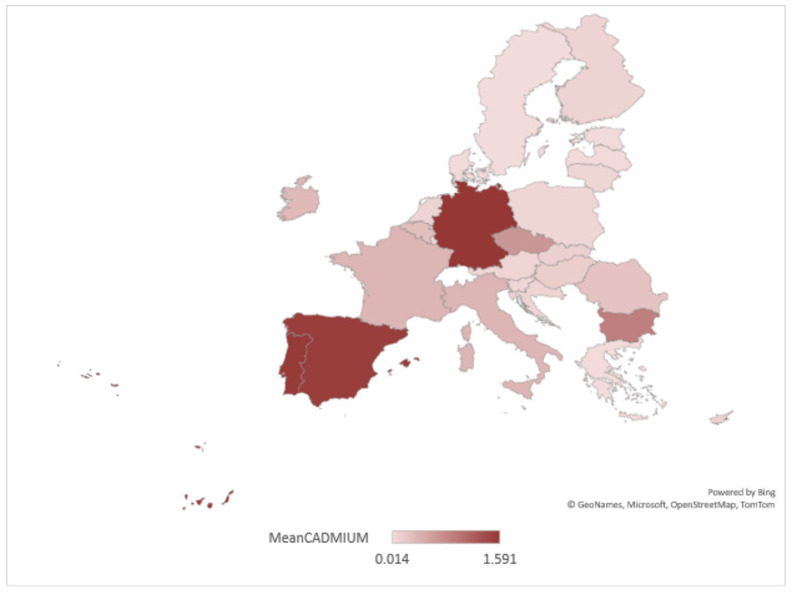
Disparities in policies to combat cadmium water pollution in the EU.

From the study carried out at the EU level, it emerged that as far as heavy metal pollution is concerned, there have been sustained reductions in pollution levels since 2000, both through the creation of an appropriate legislative framework to reduce pollution (UNECE, 2021, ‘Protocol on heavy metals’, United Nations Economic Commission for Europe [57] and through the issuance of European directives [58], which have allowed the stricter monitoring of and reduction in heavy metal emissions to water and air. The most notable results in this area have been recorded in the manufacturing and extractive sectors [2], but the energy sector has also contributed to the reduction in pollution, with an increase in the reduction in pollution observed during periods of economic crisis (2008 or 2020). In real terms, the reduction in cadmium pollution is mapped in Figure 7.

At the European level, according to official data, between 2000 and 2021, the highest pollution levels were recorded on average in countries such as Germany, Spain and Portugal, while the opposite was true for Greece and Sweden. As cadmium directly affects the health of the aquatic environment, in many EU member countries, increased concentration levels lead to adverse eutrophication effects. This has changed the composition of bottom communities, leading to a reduction in fish and aquatic animal populations. In the less polluted areas of the Nordic countries, cadmium concentrations are as low as 10% and have led to increased trade in fish products amid growing global consumer confidence in the health and food safety of fish products from these regions. In contrast, there has been a decline in consumer confidence in fish products from the countries most affected by cadmium pollution (species such as mussels).

**Figure 8:**
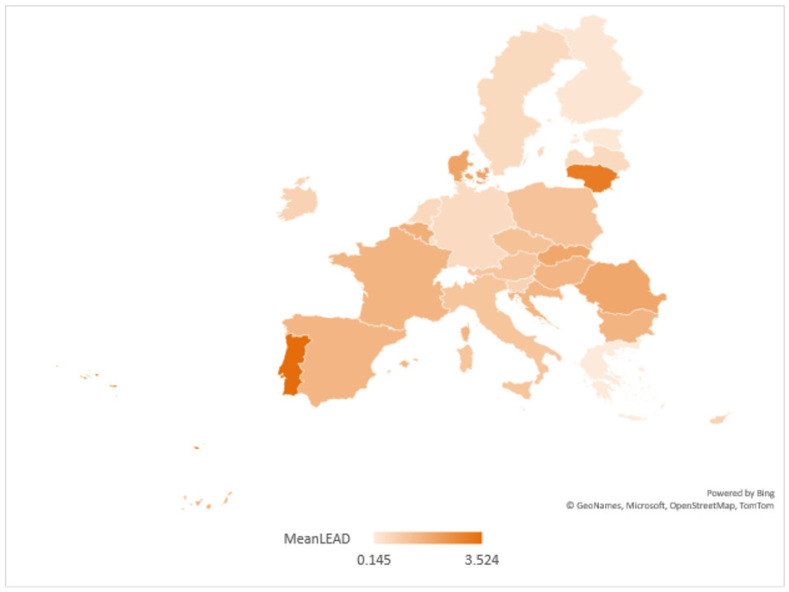
Disparities in policies to combat lead water pollution in the EU.

In the case of lead water pollution, the average pollution map based on official reports [46] is shown in Figure 8.

According to Figure 8, the most lead-polluted waters were in Lithuania, Portugal, Romania and Denmark. At the opposite pole are Estonia and Greece. In terms of pollution reduction policies, the most significant progress has been achieved in the transport sector, the use of lead-free petrol, the reduction in lead concentration in pipelines, the reduction in pesticides used in agriculture and the shift to organic farming. The effect is to mitigate the degradation of the aquatic environment and improve the quality of fish products (cod fished in the NW Atlantic and mussels fished in the NE Atlantic and Mediterranean).

**Figure 9:**
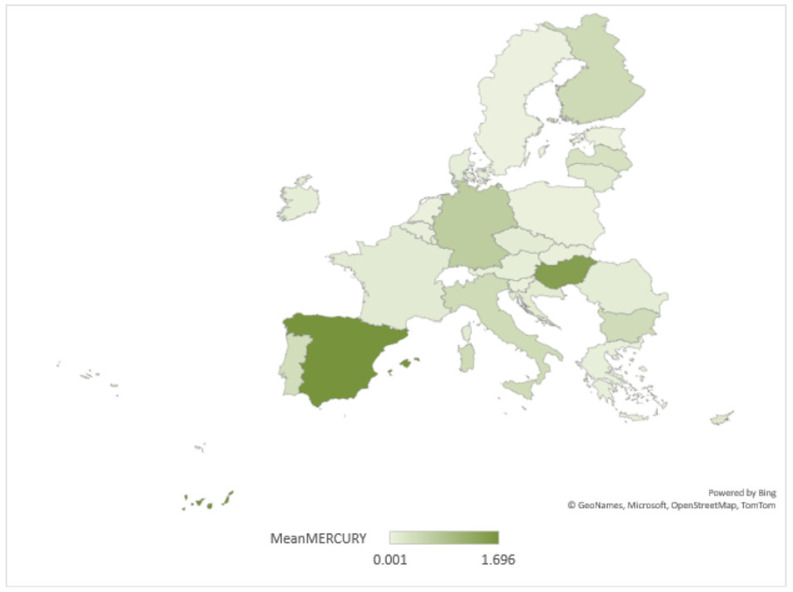
Disparities in policies to combat mercury water pollution in the EU.

Regarding mercury pollution, the results of the study are shown in Figure 9.

According to Figure 9, the most mercury-polluted waters were in Spain and Hungary. At the opposite pole are Estonia, the Netherlands and Sweden. Europe has been a major user and emitter of mercury, but significant legislative efforts over the last 40 years have substantially reduced use and emissions to the environment.

At present, we are witnessing the fading of the degradation of the aquatic environment and the improvement of the quality of fish products (mussels caught in the NE Atlantic and Mediterranean, large predatory fish (tuna, swordfish) and seafood). It is estimated that in Europe alone, more than 1.8 million babies are born each year with mercury levels above recommended safe limits [59].

The research carried out an analysis of the impact of non-consolidated pollution reduction policies, showing that although general objectives have been agreed at the European level, there is a wide disparity in the level of reduction in heavy metal water pollution in the member states.

Based on these disparities, the following framework presented in Table 4 is highlighted as a general framework of vulnerabilities in combating heavy metal water pollution.

The proposed policies in Table 4 may constitute future directions for the development of heavy metal pollution control in European and world waters. These policies can be linked to the current action guidelines adopted by supranational bodies in the field of pollution control.

## 5. Conclusions

The analysis of the status of heavy metal water pollution in the EU carried out by the authors in order to identify a new general model of pollution achieved the proposed objectives in terms of identifying the dynamics of heavy metal pollution at the European level, the authors mapping for the three elements studied (cadmium, lead, mercury) the status of pollution with examples of the implications and the level of environmental policies dedicated to them.

Through this research, the general spatio-temporal model of combating the heavy metal pollution of inland, coastal and marine waters was defined. This model allows to capture the spatio-temporal water pollution matrix with the application of the contributive criterion, resulting in a general model that captures the evolution of combined policies to reduce heavy metal pollution in European waters.

Compared with the analysed literature in the Literature review section, this article may have these marginal contributions concerning the definition, testing and implementation of the general model to combat heavy metal pollution in the EU in a scientific manner based on official statistical reports and the authors’ consolidated database.

The novelty of the study lies in the new spatio-temporal approach to the issue of heavy metal water pollution, including the design of the spatio-temporal matrix and its related model, which have been exploited by the authors through the concrete identification of vulnerabilities and the formulation of policy proposals in the field of combating heavy metal water pollution.

Quantifying the contribution of regional policies in the field to the overall pattern and mapping the levels of public policy disparities at the European level require the identification of effective solutions to strengthen the fight against heavy metal water pollution in Europe.

The main limitations of the study are the relatively limited number of heavy metals taken into analysis. In addition, the data collected by the authors come from European Environment Agency sources and cover only member states. The findings of the study can be extended to other European countries that are not members of the EU but benefit from the same river basins. The authors aim to broaden the study by completing the sample of heavy metals analysed and strengthening regional water pollution control policies.

## Figures and Tables

**Table 1:** Technical data on analysed indicators.

Monitoring Country	Dataset Monitoring	Water Body Category	Observed Property Determinant Code	Observed Property Determinant Label	Procedure Analysed Matrix	Result m.u.
AT-AustriaBA-SloveniaBE-BelgiumBG-BulgariaCY-Cyprus CZ-Czechia DE-Germany EE-Estonia EL-Greece ES-Spain FI-Finland FR-France HR-Croatia HU-Hungary IE-Ireland IT-Italy LT-Lithuania LU-Luxembourg LV-Latvia MT-Malta NL-Netherlands PL-Poland PT-Portugal RO-Romania SE-Sweden SK-Slovakia	WISE SoE—Water Quality in Inland, Coastal and Marine waters (WISE-6)	All water body categories	CAS_7440-43-9	Cadmium and its compounds	Aggregateddata	μg/L
			CAS_7439-97-6	Mercury and its compounds		
			CAS_7439-92-1	Lead and its compounds		

**Table 2:** Results of econometric correlations of spatio-temporal heavy metal pollution at Member State level.

Country	Correlations CADMIUM-LEAD	Std. Error CADMIUM-LEAD	*t*-Ratio CADMIUM-LEAD	*p*-Value CADMIUM-LEAD	Sig. CADMIUM-LEAD	Correlations CADMIUM-MERCURY	Std. Error CADMIUM-MERCURY	*t*-Ratio CADMIUM-MERCURY	*p*-Value CADMIUM-MERCURY	Sig. CADMIUM-MERCURY	Uncentered R-Squared
Austria	0.049	0.007	7.001	<0.0001	***	0.432	0.137	3.161	0.0049	***	0.963
Slovenia	0.051	0.025	2.058	0.0529	*	0.679	0.834	0.815	0.4249		0.574
Belgium	0.189	0.083	2.265	0.0347	**	1.557	2.605	0.598	0.5567		0.584
Bulgaria	−0.619	0.208	−2.981	0.0074	***	4.946	0.890	5.560	<0.0001	***	0.666
Cyprus	−0.003	0.023	−0.124	0.9023		0.483	0.074	6.492	<0.0001	***	0.841
Czechia	0.433	0.248	1.746	0.0962	*	0.190	2.429	0.078	0.9386		0.374
Germany	0.763	0.340	2.242	0.0365	**	1.343	0.209	6.438	<0.0001	***	0.750
Estonia	−0.072	0.025	−2.893	0.009	***	2.453	0.314	7.817	<0.0001	***	0.988
Greece	0.077	0.029	2.638	0.0158	**	0.334	0.172	1.946	0.0658	*	0.528
Spain	1.690	0.204	8.282	<0.0001	***	−0.553	0.164	−3.372	0.003	***	0.832
Finland	0.072	0.013	5.655	<0.0001	***	0.196	0.006	30.580	<0.0001	***	0.986
France	0.080	0.060	1.334	0.1973		2.201	0.886	2.484	0.022	**	0.722
Croatia	0.052	0.028	1.877	0.0752	*	−0.264	0.311	−0.848	0.4066		0.319
Hungary	0.149	0.008	18.020	<0.0001	***	−0.057	0.008	−7.458	<0.0001	***	0.952
**Ireland**	**0.331**	**0.161**	**2.053**	**0.0534**	*	**1.014**	**1.777**	**0.571**	**0.5747**		**0.934**
Italy	0.126	0.065	1.930	0.0679	*	0.193	0.156	1.231	0.2325		0.584
Lithuania	0.009	0.005	1.832	0.0819	*	0.321	0.092	3.482	0.0024	***	0.957
Luxembourg	−0.034	0.004	−9.194	<0.0001	***	0.413	0.012	35.390	<0.0001	***	0.997
Latvia	0.059	0.011	5.612	<0.0001	***	−0.023	0.019	−1.242	0.2287		0.754
Malta	−0.017	0.057	−0.299	0.7679		12.623	1.575	8.013	<0.0001	***	0.832
Netherlands	−0.032	0.032	−1.004	0.3274		6.577	0.831	7.911	<0.0001	***	0.829
Poland	0.148	0.038	3.857	0.001	***	−4.645	2.038	−2.279	0.0338	**	0.854
Portugal	0.600	0.030	20.010	<0.0001	***	−1.461	0.181	−8.071	<0.0001	***	0.952
Romania	0.105	0.017	6.286	<0.0001	***	0.289	0.301	0.959	0.3491		0.809
Sweden	0.030	0.005	5.945	<0.0001	***	−2.813	2.843	−0.990	0.3342		0.842
Slovakia	0.067	0.011	5.995	<0.0001	***	−0.564	0.527	−1.070	0.2972		0.748
Denmark	0.007	0.001	7.288	<0.0001	***	0.271	0.027	10.080	<0.0001	***	0.985

*- medium level of statistical significance; **- high level of statistical significance; ***- highest level of statistical significance. **Ireland—optimal model configuration**.

**Table 3:** European model for the management of heavy metal water pollution 2000–2021.

Indicator	Coefficient	Std. Error	*t*-Ratio	*p*-Value	Sig.
EUMeanLEAD	0.331254	0.161347	2.053	0.0534	*
EUMeanMERCURY	1.01366	1.77685	0.5705	0.5747	
Mean-dependent var	0.340682	S.D.-dependent var		0.254302	
Sum squared resid	0.259830	S.E. of regression		0.113980	
Uncentred R-squared	0.933572	Centred R-squared		0.808675	
F(2, 20)	140.5393	*p*-value(F)		1.67 × 10^−12^	
Log-likelihood	17.60982	Akaike criterion		−31.21963	
Schwarz criterion	−29.03755	Hannan–Quinn		−30.70560	
Spearman Correlation (rho)	0.630820	Durbin–Watson		0.698846	
Test for normality of residual:		Breusch-Pagan test for heteroskedasticity			
Null hypothesis: error is normally distributed		Null hypothesis: heteroskedasticity not present			
Test statistic: Chi-square(2)	3.54861	Test statistic: LM		7.43767	
with *p*-value	0.169601	with *p*-value = P(Chi-square(2) > 7.43767)		0.0242622	

* Based on statistical data reported by European Environment Agency. URL http://discomap.eea.europa.eu/data/wisesoe/deriveddata/T_WISE4_AggregatedDataByWaterBody/0.html (accessed on 10 October 2022).

**Table 4:** General framework of vulnerabilities on combating heavy metal pollution in Europe.

Type of Element	Vulnerabilities Regarding Pollution Control	Effects of Vulnerabilities	Policy Proposals to Improve Water Pollution Control Measures
Cadmium, Mercury, Lead	Differentiated national pollution reduction policies.	Increasing population health security disparities; differential reduction in aquatic ecosystem health; degradation of aquatic ecoregions.	Establishing a new pollution control index; better informing the population through public alerts in case the critical water pollution index is exceeded; establishment of monitoring points for the quality of aquatic products marketed for consumption; cross-border cooperation in the field.
	Impact of the energy crisis on heavy metal water pollution	Following the outbreak of geo-political conflict, the EU was faced with the need to identify alternative sources of energy, including energy based on solid fuels. This has led to the reopening of some mining operations, which, due to their location near water sources, have increased the risk of heavy metal pollution.	In this context, the careful monitoring of lignite and hard coal mining operations is needed, requiring good planning of activities, environmental impact and soil condition analyses and water pollution variation in the region.
	Demographic dynamics and impact on heavy metal water pollution	Urban agglomerations increase the risk of water pollution. Thus, the quality of drinking water in these agglomerations is closely related to the health of the population.	The European Environment Agency needs to expand the set of core indicators for water to help coordinate European water reporting activities and make them more policy-relevant. Strengthening the Eurowaternet network for collecting data on the quantitative and qualitative status of water waste and its impact on the environment.
	Changing climatic conditions and impact on heavy metal water pollution	In the current context, climate transformation has the effect of changing the precipitation regime and altering the circulation of dust in the atmosphere, while decreasing the natural capacity of water to regenerate.	Implementation of an online climate change impact map, highlighting possible phenomena (rainfall, floods, droughts, dust transport from Saharan areas or other areas of the globe) that can be linked to the water pollution monitoring network and issue warnings on potential risks of increased water pollution.

## Data Availability

The data presented in this study are available on request from the corresponding author.
